# The performance of different propensity score methods for estimating marginal hazard ratios

**DOI:** 10.1002/sim.5705

**Published:** 2012-12-12

**Authors:** Peter C Austin

**Affiliations:** a Institute for Clinical Evaluative SciencesToronto, Ontario, Canada; b Institute of Health Management, Policy and Evaluation, University of TorontoToronto, Ontario, Canada; c Dalla Lana School of Public Health, University of TorontoToronto, Ontario, Canada

**Keywords:** propensity score, survival analysis, inverse probability of treatment weighting (IPTW), Monte Carlo simulations, observational study, time-to-event outcomes

## Abstract

Propensity score methods are increasingly being used to reduce or minimize the effects of confounding when estimating the effects of treatments, exposures, or interventions when using observational or non-randomized data. Under the assumption of no unmeasured confounders, previous research has shown that propensity score methods allow for unbiased estimation of linear treatment effects (e.g., differences in means or proportions). However, in biomedical research, time-to-event outcomes occur frequently. There is a paucity of research into the performance of different propensity score methods for estimating the effect of treatment on time-to-event outcomes. Furthermore, propensity score methods allow for the estimation of marginal or population-average treatment effects. We conducted an extensive series of Monte Carlo simulations to examine the performance of propensity score matching (1:1 greedy nearest-neighbor matching within propensity score calipers), stratification on the propensity score, inverse probability of treatment weighting (IPTW) using the propensity score, and covariate adjustment using the propensity score to estimate marginal hazard ratios. We found that both propensity score matching and IPTW using the propensity score allow for the estimation of marginal hazard ratios with minimal bias. Of these two approaches, IPTW using the propensity score resulted in estimates with lower mean squared error when estimating the effect of treatment in the treated. Stratification on the propensity score and covariate adjustment using the propensity score result in biased estimation of both marginal and conditional hazard ratios. Applied researchers are encouraged to use propensity score matching and IPTW using the propensity score when estimating the relative effect of treatment on time-to-event outcomes. Copyright © 2012 John Wiley & Sons, Ltd.

## 1. Introduction

Observational studies are increasingly being used to estimate the effects of treatments, interventions, and exposures on outcomes. These studies allow for the examination of treatment effects in settings in which randomized controlled trials (RCTs) may be unethical or impractical. Furthermore, observational studies allow for the study of treatment efficacy outside of the tightly controlled environment of an RCT, allow for the inclusion of subjects who may have been excluded from RCTs, and allow for the study of rare outcomes and adverse events for which RCTs may have inadequate statistical power. The advantage of RCTs is that random allocation of treatment assignment allows one to obtain an unbiased estimate of the average treatment effect 1. This is because there will, on average, be no systematic differences in baseline covariates between treatment groups. In contrast, in observational studies, treatment allocation is frequently influenced by subject characteristics. Therefore, there often exist systematic differences between treatment groups in observational studies. We must use statistical methods to remove or minimize the effect of this confounding so that valid inferences on treatment effect can be drawn from observational studies.

Propensity score methods are increasingly being used to reduce or minimize the confounding that occurs frequently in observational studies of the effect of treatment on outcomes. The propensity score is the probability of treatment assignment conditional on measured baseline covariates 2. There are four ways of using the propensity score to reduce confounding: matching on the propensity score, stratification on the propensity score, inverse probability of treatment weighting (IPTW) using the propensity score, and covariate adjustment using the propensity score. These methods are often used in the biomedical literature 3,4.

Conditioning on the propensity score results in unbiased estimates of linear treatment effects 2. Thus, when outcomes are continuous, conditioning on the propensity score allows for unbiased estimation of differences in means. However, in biomedical research, outcomes are commonly binary or time to event in nature, rather than continuous 5. When outcomes are binary, we can estimate the effect of treatment using the risk difference (difference in proportions or absolute risk reduction) (along with the associated number needed to treat, which is the reciprocal of the absolute risk reduction), the relative risk, and the odds ratio. Several studies have examined the performance of different propensity score methods for estimating treatment effects when outcomes are binary 6–9. Although propensity score methods have frequently been used in the analysis of time-to-event outcomes, there is a paucity of research examining the relative performance of different propensity score methods for estimating hazard ratios.

A conditional effect is the average effect, at the subject level, of moving a subject from untreated to treated. The regression coefficient for a treatment assignment indicator variable from a multivariable regression model is an estimate of a conditional or adjusted effect. In contrast, a marginal effect is the average effect, at the population level, of moving an entire population from untreated to treated 10. Linear treatment effects (differences in means and differences in proportions) are collapsible: the conditional and marginal treatment effects will coincide. However, when outcomes are binary or time to event in nature, the odds ratio and the hazard ratio are not collapsible 11. Rosenbaum has noted that propensity score methods allow one to estimate marginal, rather than conditional, effects 12. There is a paucity of research into the performance of different propensity score methods to estimate marginal treatment effects.

The objective of the current study is to examine the ability of different propensity score methods to estimate marginal and conditional hazard ratios when outcomes are time to event in nature. The paper is structured as follows. In Section 2, we describe different propensity score methods and how they can be used to estimate hazard ratios for survival outcomes. In Section 3, we describe the design and results of an extensive series of Monte Carlo simulations to compare the performance of different propensity score methods to estimate hazard ratios. In Section 4, we summarize our findings and place them in the context of the existing literature.

## 2. Propensity score methods and survival outcomes

We use the following notation throughout this section. Let *Z* be an indicator variable denoting treatment status (*Z* = 1 for active treatment of interest and *Z* = 0 for the control treatment), whereas *e* denotes the estimated propensity score.

### 2.1. Matching on the propensity score

Matching on the propensity score entails forming matched sets of treated and untreated subjects who have a similar value of the propensity score 13. The most common implementation of propensity score matching is pair matching or 1:1 matching in which matched pairs of treated and untreated subjects are formed. In the current study, we used greedy nearest-neighbor matching within specified caliper widths to form pairs of treated and untreated subjects matched on the logit of the propensity score 13. We used calipers of width equal to 0.2 of the standard deviation of the logit of the propensity score as this caliper width has been found to perform well in a wide variety of settings 14.

Once a propensity-score-matched sample had been formed, we estimated the effect of treatment on survival using three different methods. First, we used a Cox proportional hazards regression model to regress survival on an indicator variable denoting treatment status. We used model-based standard errors to estimate 95% confidence intervals. Second, we fitted the same model as before; however, we obtained a robust sandwich estimate of the variance of the regression coefficient that accounted for the clustering within matched sets 15. Finally, we fitted a univariate Cox model as before; however, this model stratified on matched sets, thereby allowing the baseline hazard function to vary across matched sets. We carried this out to account for the potential homogeneity of outcomes within matched sets. Cummings, McKnight, and Greenland proposed the use of stratification on matched sets to account for matched cohort designs with time-to-event outcomes 16. We will refer to these three models as the naïve Cox model, the robust Cox model, and the stratified Cox model, respectively.

### 2.2. Stratification on the propensity score

Stratification (or subclassification) on the propensity score stratifies the entire sample into mutually exclusive subclasses on the basis of the propensity score. A common approach is to define the subclasses using specified quantiles of the propensity score. Using the quintiles of the estimated propensity score to divide the sample into five, approximately equally sized, groups has been shown to eliminate approximately 90% of the bias due to measured confounding variables when estimating a linear treatment effect 2,17,18. We used stratification on the quintiles of the propensity score in the current study given its popularity in the applied literature.

When estimating a linear treatment effect (e.g., a difference in means or difference in proportions), one can estimate stratum-specific treatment effects and then pool or average these stratum-specific effects across the strata 2,17. We examined three modifications to this approach for estimating hazard ratios when outcomes are time to event in nature. Each method was based on a Cox regression model with survival as the outcome variable. In the first method, we included two explanatory variables in the Cox model: an indicator variable denoting treatment status and a categorical variable denoting propensity score strata (as a five-level categorical variable). In the second method, we fit five stratum-specific univariate Cox regression models in which survival was regressed on an indicator variable denoting treatment status. We then pooled or averaged the five log-hazard ratios to estimate an overall treatment effect. Third, we fit a univariate Cox model in which we regressed survival on an indicator variable denoting treatment status. The model stratified on the five propensity score strata, thereby allowing the baseline hazard ratio to vary across the propensity score strata. We refer to these three methods as stratification (adjusted), stratification (pooled), and stratification (stratified), respectively.

### 2.3. Inverse probability of treatment weighting using the propensity score

The IPTWs are defined as (*Z* / *e*) + [(1 − *Z*) / (1 − *e*)] 19. Weighting the sample using these weights results in a weighted synthetic sample in which observed baseline covariates are not confounded with treatment assignment. Using these weights allows one to estimate the average treatment effect (ATE). Using weights equal to *Z* + [*e*(1 − *Z*) / (1 − *e*)] allows one to estimate the average treatment effect in the treated (ATT) 20. In the weighted sample (weighted using either the ATE weights or the ATT weights), we used a Cox regression model to regress survival on an indicator variable denoting treatment status and used a robust variance estimator 15,21.

### 2.4. Covariate adjustment using the propensity score

Rosenbaum and Rubin proposed covariate adjustment using the propensity score in the context of estimating linear treatment effects for continuous outcomes 2. Using this approach, we regress the outcome on two covariates: an indicator variable denoting treatment status and the propensity score. The regression coefficient associated with the treatment selection indicator represents the effect of treatment. In the current study, we used Cox regression to regress survival time on these two variables. The regression coefficient for the treatment status indicator is the estimated log-hazard ratio.

## 3. Monte Carlo simulations

We used a series of Monte Carlo simulations to examine the relative performance of different propensity score methods to estimate hazard ratios. Our primary focus was on estimating marginal or population-average hazard ratios. However, as a secondary objective, we also examined estimation of conditional hazard ratios.

### 3.1. Monte Carlo simulations—methods

We simulated data for a setting in which there were 10 baseline covariates (*X*_1_– *X*_10_). We simulated these covariates from independent standard normal distributions. Of these 10 covariates, seven affected treatment selection (*X*_1_– *X*_7_), whereas seven affected the outcome (*X*_4_– *X*_10_). Furthermore, we allowed covariates to have a weak, moderate, strong, or very strong effect on treatment selection or outcome. For each subject, we determined the probability of treatment selection from the following logistic model: logit(*p*_*i*_) = *α*_0,treat_ + *α*_W_*x*_1_ + *α*_M_*x*_2_ + *α*_S_*x*_3_ + *α*_W_*x*_4_ + *α*_M_*x*_5_ + *α*_S_*x*_6_ + *α*_VS_*x*_7_. We selected the intercept of the treatment selection model (*α*_0,treat_) so that the proportion of subjects in the simulated sample that were treated was fixed at the desired proportion. The regression coefficients *α*_W_, *α*_M_, *α*_S_, and *α*_VS_ were set to log(1.25), log(1.5), log(1.75), and log(2), respectively. These were intended to denote weak, moderate, strong, and very strong treatment assignment affects. For each subject, we generated treatment status from a Bernoulli distribution with subject-specific parameter *p*_*i*_.

We then generated a time-to-event outcome for each subject using a data-generating process for time-to-event outcomes described by Bender *et al*. 22. For each subject, we defined the linear predictor as LP = *β*_treat_*Z* + *α*_W_*x*_4_ + *α*_M_*x*_5_ + *α*_S_*x*_6_ + *α*_VS_*x*_7_ + *α*_W_*x*_8_ + *α*_M_*x*_9_ + *α*_S_*x*_10_. For each subject, we generated a random number from a standard uniform distribution: *u* ∼ U(0,1). We generated a survival or event time for each subjects as follows: − log(*u*) / (*λe*^LP^) ^1 / *η*^. We set *λ* and *η* to be equal to 0.00002 and 2, respectively. The use of this data-generating process results in a conditional treatment effect, with a conditional hazard ratio of exp(*β*_treat_). However, we wanted to generate data in which there was a specified marginal hazard ratio. To do so, we modified previously described data-generating processes for generating data with a specified marginal odds ratio or risk difference 23, b24. We used an iterative process to determine the value of *β*_treat_ (the conditional log-hazard ratio) that induced the desired marginal hazard ratio. Briefly, using the aforementioned conditional model, we simulated a time-to-event outcome for each subject, first assuming that the subject was untreated and then assuming that the subject was treated. In the sample consisting of both potential outcomes (survival or event time under lack of treatment and survival or event time under treatment), we regressed the survival outcome on an indicator variable denoting treatment status. The coefficient for the treated status indicator denotes the log of the marginal hazard ratio. We repeated this process 1000 times to obtain an estimate of the log of the marginal hazard ratio associated with a specific value of *β*_treat_ in our conditional outcome model. We then employed a bisection approach to determine the value of *β*_treat_ that resulted in the desired marginal hazard ratio. We applied this process twice: first to determine the value of *β*_treat_ that induced a desired marginal hazard ratio in the overall population. We will describe this as the ATE in the population. Second, we repeated the process but used only subjects who were ultimately assigned to the treatment when estimating the marginal hazard ratio (i.e., we fit the Cox model on the dataset of potential outcomes restricted to those subjects who were ultimately treated). We thus determined the value of *β*_treat_ that induced a desired marginal hazard ratio in the treated population. We describe this as the ATT. We acknowledge that describing these hazard ratios as average treatment effects is a slight abuse of convention as they do not explicitly involve taking expectations of differences in potential outcomes. However, our intent was to describe the average effect in the entire population or in the population of treated subjects.

We allowed the following factors to vary in our Monte Carlo simulations: the percentage of subjects that were treated (5%, 10%, and 25%) and the true marginal hazard ratio (0. 8, 1, 1. 10, 1. 25, 1. 50, 1. 75, and 2). We thus examined 21 scenarios (three treatment prevalences × seven marginal hazard ratios). For each true marginal hazard ratio, we considered both the ATE and ATT hazard ratios. In each scenario, we simulated 10,000 datasets, each consisting of 10,000 subjects.

Within each simulated dataset, we did the following: we estimated the propensity score using a logistic regression model to regress treatment status on the seven baseline covariates that affected the outcome. We selected this approach to variable selection for the propensity score model, as it has been shown to result in better estimation compared with selecting only those variables that affect treatment selection 25.

In each of the 10,000 simulated datasets for each scenario, we estimated the log-hazard ratio and its standard error using the methods described in Section 2. Let θ_*i*_ denote the estimated log-hazard ratio obtained from the *i*th simulated dataset using a given method, whereas θ denotes the true log-marginal hazard ratio. We estimated the mean treatment effect (on the log-hazard scale), bias, and mean squared error (MSE) as 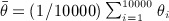, 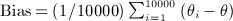, and 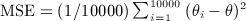, respectively. We defined relative bias as 100 × (Bias / *θ*). We also examined the accuracy with which the estimated standard error of the estimated log-hazard ratio estimated the sampling variability of the estimated log-hazard ratio. To do so, we compared two estimates. First, within each of the 10,000 simulated datasets, we estimated the standard error of estimated log-hazard ratio; we then determined the mean standard error of the log-hazard ratio across the 10,000 simulated datasets. Second, we determined the standard deviation of the estimated log-hazard ratios across the 10,000 simulated datasets. The first quantity estimates the mean standard error, whereas the second quantity estimates the sampling variability of the log-hazard ratio. We then determined the ratio of these two quantities. If the ratio equals 1, then the estimated standard error of the log-hazard ratio is correctly estimating the sampling variability of the estimated log-hazard ratio. Finally, within each simulated dataset and for each method, we computed the 95% confidence interval for the estimated hazard ratio. We then determined the mean length of the estimated 95% confidence intervals as well as the proportion of 95% confidence intervals that covered the true hazard ratio that was used in the data-generating process.

### 3.2. Monte Carlo simulations—results

When the proportion of subjects who were treated was 0.05, 0.10, and 0.25, then the average number of matched pairs formed across the 10,000 simulated samples for each scenario was 499.9, 993.4, and 2357.4, respectively. Thus, we matched approximately 100%, 99.3%, and 94.3% of treated subjects to an untreated subject. Thus, the matched methods should have minimal bias due to incomplete matching 13.

**Figure 1:**
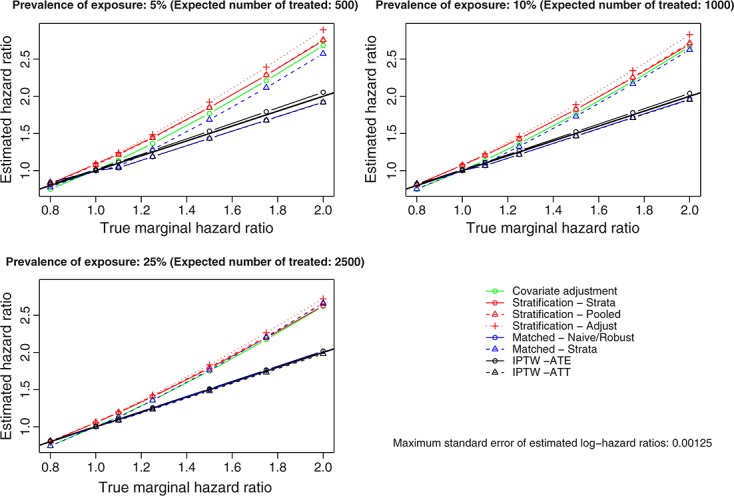
Estimated hazard ratio using different propensity score methods. IPTW, inverse probability of treatment weight; ATE, average treatment effect; ATT, average treatment effect in the treated.

**Figure 2:**
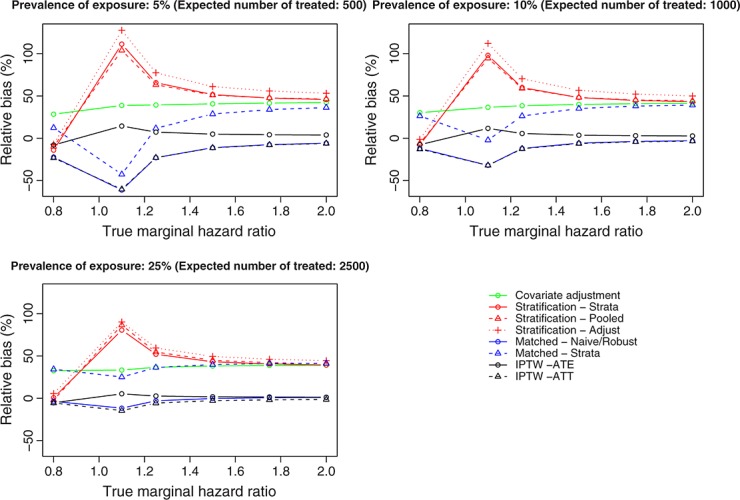
Relative bias of different propensity score methods for estimating marginal hazard ratios. IPTW, inverse probability of treatment weight; ATE, average treatment effect; ATT, average treatment effect in the treated.

Figure 1 shows the exponential of the mean estimated treatment effect 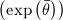, whereas Figure 2 shows the relative bias in estimating the log-marginal-hazard ratios (note that the relative biases are not reported for the scenarios with a true hazard ratio of 1 as the log-hazard ratio is 0). In Figure 1, we have added a solid diagonal line of unit slope. Deviation from this diagonal line indicates biased estimation of marginal hazard ratios. The use of 10,000 simulated datasets resulted in precise estimation of the log-hazard ratio. Across the 21 scenarios and the different estimation methods, the maximum standard error of the estimated log-hazard ratio was 0.00125. Both bias and relative bias tended to be substantial for covariate adjustment using the propensity score and for the three stratification methods. When we use matching, the naïve and robust methods resulted in the same relative bias as they are both marginal models of the same functional form and resulted in the same estimate of the regression coefficient for the log-hazard ratio. The method that stratified on matched pairs resulted in substantially greater bias than did the other two matching methods. IPTW, using either set of weights, and two of the matching methods (naïve and robust) resulted in estimates with minimal bias.

The preceding results identify several issues that must be addressed before proceeding to examination of further results. The naïve Cox model and the robust Cox model in the propensity-score-matched sample are marginal models—they estimate population-average effects. Similarly, the IPTW method estimates marginal effects. The high biases observed for covariate adjustment using the propensity score, stratification using the propensity score, and the Cox model that stratified on matched pairs in the propensity-score-matched sample highlight that these methods do not estimate marginal effects. Instead, they result in conditional measures of effect—they estimate conditional hazard ratios. That this is true for covariate adjustment using the propensity score is obvious, and it did not require simulations to illustrate this fact. That it is true for the different stratified approaches may be less obvious. With the stratification (adjusted) approach, one is fitting a conditional model that estimates the effect of treatment selection after *adjusting* for the propensity score strata (as a categorical variable). The stratification (strata) approach fits a univariate Cox model that stratifies on the propensity score strata, thereby allowing the baseline hazard function to vary across the propensity score strata. However, one is still deriving estimates of treatment effect from a model that conditions on the strata. The stratification (pooled) approach fits stratum-specific univariate Cox models. However, one then pools the stratum-specific hazard ratios. Pooling or averaging these stratum-specific estimates results in a smoothed estimate that represents an adjusted or conditional effect. Thus, none of these approaches allows one to estimate a marginal hazard ratio. Similarly, the model fit in the propensity-score-matched sample that stratified on the matched pairs is a conditional model that conditions on the matched pairs. Thus, none of these methods results in marginal estimates of treatment effect.

**Figure 3:**
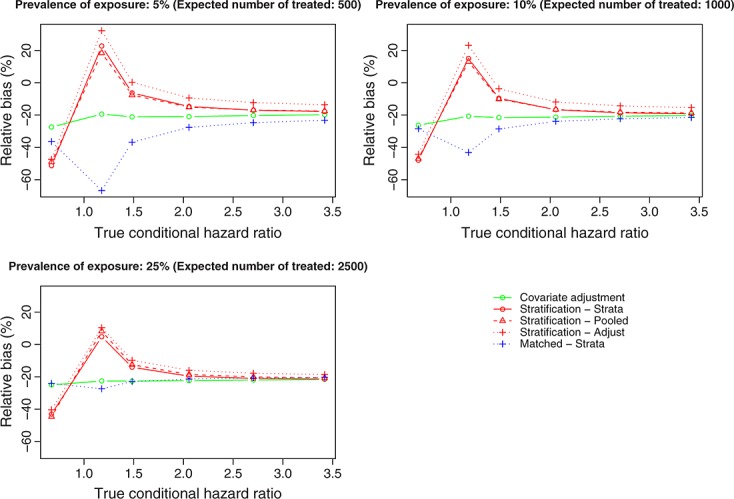
Relative bias of different propensity score methods for estimating conditional hazard ratios.

Our data-generating process employed a conditional Cox model to generate outcomes. We selected the conditional log-hazard ratio so as to induce a specified marginal hazard ratio. We examined the relative bias of covariate adjustment using the propensity score, stratification on the propensity score, and the matched analysis that stratified on matched pairs when estimating the underlying conditional hazard ratio that was used in the data-generating process. Because this set of propensity score methods resulted in biased estimation of the underlying marginal hazard ratio, it is important to examine whether this set of methods also result in biased estimation of the conditional hazard ratio used in the data-generating process. Figure 3 shows the relative bias for estimating the conditional hazard ratio. Each method resulted in moderate bias in estimating the conditional hazard ratio used in the data-generating process. In theory, there are multiple conditional effects. Indeed, there is potentially a different conditional effect for every set of covariates for which one adjusts in a regression model. Thus, although these methods allow estimation of conditional effects, it is unclear which conditional effect they are estimating. In particular, the conditional effect that is being estimated does not appear to coincide with the conditional effect employed in the data-generating process.

For the reasons noted earlier, for the remainder of this section, we focus on the performance of the two IPTW methods (ATE and ATT weights), the naïve Cox model in the propensity-score-matched sample, and the robust Cox model in the propensity-score-matched sample for estimating marginal hazard ratios.

**Figure 4:**
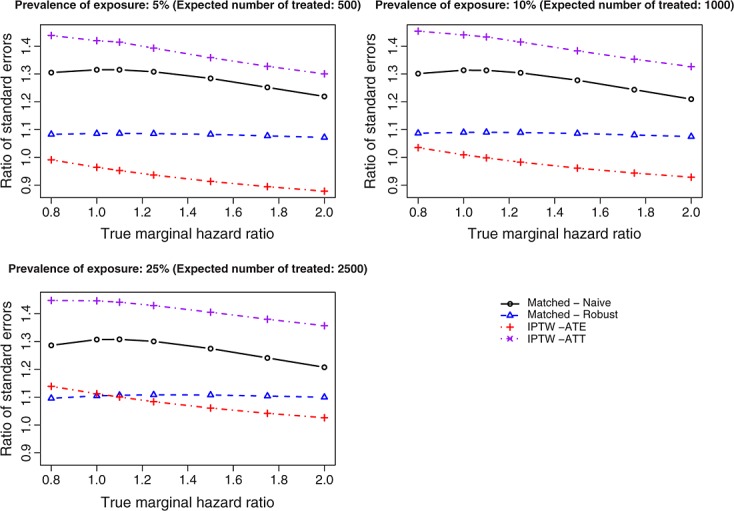
Ratio of mean standard error to standard deviation of estimated log-hazard ratios. IPTW, inverse probability of treatment weight; ATE, average treatment effect; ATT, average treatment effect in the treated.

In Figure 4, we report, for each estimation method, the ratio of the mean standard error of the estimated log-hazard ratio across the 10,000 simulated datasets (i.e., in each simulated dataset, we obtained an estimated standard error of the estimated treatment effect; we then averaged these across the 10,000 simulated datasets) to the standard deviation of the estimated log-hazard ratios across the 10,000 simulated datasets (i.e., in each dataset, we obtained an estimated log-hazard ratio; we then estimated the sampling variability of these estimated log-hazard ratio). This analysis indicates whether, for a given estimation method, the estimated standard error of the estimated treatment effect is correctly estimating the sampling variability of the estimated treatment. This ratio tended to be larger for the naïve Cox model in the matched sample than for the robust Cox model in the matched sample. The mean ratio for the naïve matched method was 1.28 across the 21 scenarios, whereas it was 1.09 for the robust matched method. Thus, ignoring the matched nature of the propensity-score-matched sample resulted in estimates of the standard error of the log-hazard ratio that was inflated by an average of 28% (i.e., the naïve matched method resulted in estimates of standard error that were, on average, 28% too large, whereas the robust matched approach resulted in estimates of standard error that were, on average, 9% too large). The mean ratios for the IPTW methods were 1.0 and 1.40 when using ATE and ATT weights, respectively.

**Figure 5:**
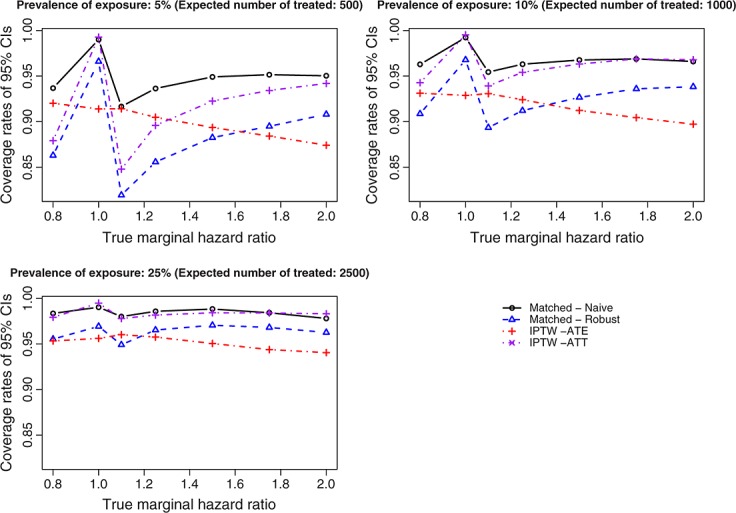
Coverage rates of 95% confidence intervals (CIs) for estimated hazard ratios. IPTW, inverse probability of treatment weight; ATE, average treatment effect; ATT, average treatment effect in the treated.

**Figure 6:**
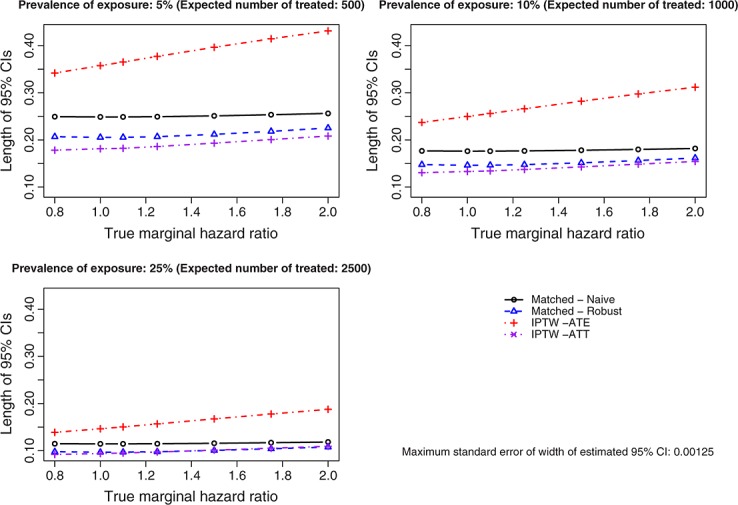
Mean length of 95% confidence intervals (CIs) for marginal hazard ratios. IPTW, inverse probability of treatment weight; ATE, average treatment effect; ATT, average treatment effect in the treated.

Figure 5 shows coverage rates of 95% confidence intervals. Because of our use of 10,000 iterations per scenario in our Monte Carlo simulations, any confidence intervals whose empirical coverage rate is less than 0.9457 or greater than 0.9543 would be statistically significantly different from 0.95, using a standard test based on the normal approximation to the binomial distribution. In general, coverage rates of 95% confidence intervals were suboptimal. Coverage rates of 95% confidence intervals tended to improve as the proportion of subjects who were treated increased. Figure 6 shows the mean length of the estimated 95% confidence intervals. Among the three methods that estimated the ATT, the naïve matched approach tended to result in estimated 95% confidence intervals that were slightly wider than those from the other two approaches. The differences between these three methods decreased as the proportion of subjects who were treated increased.

**Figure 7:**
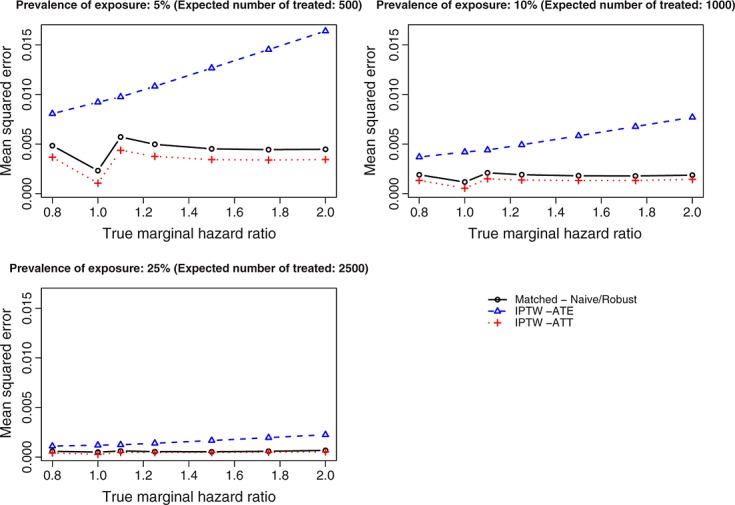
Mean squared error of estimated log-hazard ratio. IPTW, inverse probability of treatment weight; ATE, average treatment effect; ATT, average treatment effect in the treated.

Figure 7 presents the MSE of the estimated treatment effects. IPTW using the ATE weights is the only method that allows one to estimate an ATE effect. Therefore, we do not compare the performance of this method with that of the other methods, as the other methods are estimating a different quantity (the marginal effect of treatment in the treated population). However, we note that with the IPTW-ATE approach, the MSE increased as the true marginal hazard ratio increased. Because the naïve Cox model and the robust Cox model fits in the propensity-score-matched sample produce the same point estimate of the log-hazard ratio, these two methods result in estimates with the same MSE. In all 21 of the scenarios, the IPTW (ATT weights) estimator had lower MSE than did the matched estimator. Across the 21 scenarios, the mean MSE of the matched estimates was 0.0023, whereas it was 0.0017 for the IPTW (ATT) estimates. However, differences between these two approaches decreased as the proportion of subjects that were treated increased.

## 4. Discussion

We conducted an extensive series of Monte Carlo simulations to examine the performance of different propensity score methods to estimate marginal hazard ratios. We briefly summarize our findings and place them in the context of the existing literature.

Greenland and others distinguished between measures of effect that are collapsible and those that are non-collapsible 10. A measure of effect is collapsible if, in the absence of confounding, the conditional effect and the marginal effect coincide. Differences in means and risk differences are collapsible, whereas odds ratios and hazard ratios are non-collapsible 10,11. Neuhaus *et al*. demonstrated that, in general, the marginal odds ratio will be closer to the null than the conditional effect 26. Similarly, Pocock *et al*. commented that in RCTs, the adjusted (i.e., conditional) odds ratio or hazard ratio will be further from the null than the unadjusted (e.g., marginal) estimate 27. Stratification on the propensity score and covariate adjustment using the propensity score reduce bias when estimating linear treatment effects 2, which are collapsible. The current study found that these methods do not perform well when outcomes are time to event in nature and when the hazard ratio is used as the measure of effect. Both of these methods resulted in biased estimation of the marginal hazard ratio. Furthermore, they also resulted in biased estimation of the conditional hazard ratio that underlay the data-generating process. These two methods are likely estimating a conditional effect; however, it is unclear which conditional effect it is. It does not appear to be the conditional effect that would be obtained by specifying the outcome regression model correctly and including the covariates that are predictive of the outcome.

When outcomes are time to event in nature, we can summarize the effect of treatment in at least three ways: first, the hazard ratio describing the relative effect of the treatment on the hazard of the outcome; second, the estimation of Kaplan–Meier survival curves in each treatment group, which allows for estimating the absolute effect of treatment on the probability of the event occurring within any specified duration of follow-up; third, mean or expected survival can be estimated in each treatment group, and the effect of treatment on mean survival can be reported. Of these three methods, the first provides a relative measure of effect, whereas the latter two provide absolute measures of effect. The first two are the most common in reports of RCTs, whereas the latter is infrequently used in RCTs 5. In the current study, we have focused on the estimation of hazard ratios, in part, because of the frequency with which they are reported in the medical literature. There are different ways in which survival curves can be estimated and compared using propensity score methods. In a propensity-score-matched sample, we can estimate Kaplan–Meier estimates of survival curves in each treatment group separately. We have removed confounding by design: the distribution of baseline covariates will be similar between treated and untreated subjects in the matched sample. Thus, the use of the ‘crude’ Kaplan–Meier estimator can allow for an unbiased comparison of survival between treatment groups. Because of the lack of independence between the two matched samples, the log-rank test should not be used for testing the equality of the survival curves 28. Both Cole and Hernan 29 and Xie and Liu 30 have described methods to estimate survival curves in the sample weighted by the inverse of the probability of treatment. The performance of different propensity score methods for estimating the latter two measures of effect should be examined in subsequent research.

There are certain limitations to the current study. Our findings were based on an extensive series of Monte Carlo simulations. As such, our findings warrant replication in different scenarios and under different assumptions about the distribution of baseline covariates and about the number of measured baseline covariates and their relationship with treatment selection and with outcome. However, given our focus on estimating marginal hazard ratios, analytic determination of the performance of estimation would be very difficult, particularly for the methods based on propensity score matching. Furthermore, we would note that several prior studies examining the performance of propensity score methods for estimating treatment effects have employed Monte Carlo simulations 6–9,31,32. We speculate that our findings will generalize to other combinations of baseline covariates. A second limitation relates to our focus on 1:1 matching on the propensity score, in which pairs of treated and untreated subjects were formed. We did not consider *M*:1 matching in which *M* untreated subjects are matched to each treated subject. *M*:1 matching was examined in a prior publication, in which it was found that, in many scenarios, use of *M* = 1 or 2 tended to be optimal 33. Increasing the number of untreated subjects matched to each treated subject will, on average, tend to result in the matching of increasingly dissimilar subjects. This can result in increased bias in the estimated treatment. However, there will tend to be a commensurate increase in precision of the estimated treatment effect. In the current study, we found that the robust matching approach and the IPTW (ATT weights) tended to result in estimates with comparable bias. We speculate that matching multiple untreated subjects to each treated subjects would, on average, result in increased bias compared with the weighted estimator. A third limitation is our inclusion of only a single matching algorithm: greedy nearest-neighbor matching on the logit of the propensity score using calipers defined by the variance of the logit of the propensity score. This approach was included as it has been found to perform well compared with other commonly used alternatives 34. However, we did not examine other approaches such as optimal matching because of its increased computational complexity 35. It is possible that the use of optimal matching may result in improved performance of the matching approach compared with the other propensity score approaches.

The findings of the current study complement those of previously published studies. Our examination of the estimation of marginal hazard ratios using propensity score matching confirmed the observation made in prior studies that variance estimation should account for the matched nature of the sample 28,36. In particular, the use of a robust variance estimator resulted in estimates of standard error that tended to better reflected the sampling distribution of the estimated log-hazard ratio than did the use of naïve model-based standard errors from a maximum likelihood model that assumed independent observations. In a recently published study, Gayat *et al*. examined the performance of different implementations of propensity score matching and covariate adjustment using the propensity score for estimating hazard ratios 37. When using propensity-score matching, they examined a variety of estimators in the propensity-score matched sample: a crude (unadjusted) estimator, a crude (stratified) estimator, and an estimator that was adjusted for all of the measured covariates. Similar to the current study, they found that, in the matched sample, it was preferable to use a robust variance estimator, rather than the naïve variance estimator. The current study examined IPTW methods and stratification on the propensity score, which were not considered in the study by Gayat *et al*. The conclusions of these two studies differed in one respect: they found that the stratified matched approach and covariate adjustment using the propensity score resulted in at most modest bias when estimating *conditional* hazard ratios (i.e., relative biases of less than 10%), whereas we found larger biases when estimating conditional hazard ratios. We speculate that are several potential explanations for these differences: first, in the current simulations, covariates were generated from normal distributions, whereas in the paper by Gayat *et al*., they were simulated from Bernoulli distributions. Second, while the earlier paper only considered three hazard ratios, we considered seven hazard ratios in the current study. The fact that we observed moderate biases when estimating conditional hazard ratios indicates that, in general, propensity score methods result in biased estimation of the underlying conditional hazard ratio. However, the degree of the bias will vary across different settings.

The finding that propensity-score methods result in confidence intervals whose empirical coverage rates differ from the advertised levels warrants concern. This finding is similar to observations made in an earlier study comparing different propensity score methods for estimating absolute risk reductions or differences in proportions 9. We speculate that the sub-optimal coverage rates are related to estimation of the standard error of the estimated log-hazard ratio, rather than to bias in the estimated log-hazard ratio. As illustrated in Figure 1, matching on the propensity score and IPTW result in at most minimal bias in the estimation of the true log-hazard ratio. However, as illustrated in Figure 4, both methods result in mis-estimation of the variance of the sampling distribution of the estimated log-hazard ratio. Since the confidence interval is constructed using the estimated log-hazard ratio and the estimated standard error of the log-hazard ratio, it appears likely that the sub-optimal coverage rates relates to the mis-estimation of the standard error of the log-hazard ratio. Reasons for why this occur merit further investigation. One avenue that warrants exploration is the use of estimated versus known propensity scores. Propensity score methods rely on estimation of the propensity score. However, once the propensity score is estimated, the analyst acts as though it was a known quantity, rather than an estimate of an unknown quantity. Future research is required on methods for variance estimation that account for the fact that the propensity score has been estimated.

In summary, researchers should employ propensity score matching and inverse probability of treatment weighting using the propensity score when estimating the relative effect of treatment on time-to-event outcomes using observational or non-randomized data. Use of these methods allows for estimation of the marginal effect of treatment on survival—the same metric that is reported in reports of RCTs with time-to-event outcomes. An advantage to the use of IPTW using the propensity score is that by using different weights, one can estimate both ATE and ATT. While both matching and IPTW resulted in estimates of marginal hazard ratios with minimal bias, the latter approach resulted in estimates with lower MSE, suggesting that the estimates resulting from weighted analyses have improved precision than those resulting from matched analyses.
